# Benign Fibroepithelial Polyp of Renal Pelvis in a Patient with Familial Adenomatous Polyposis: A Successful Percutaneous Nephroscopic Management Strategy

**DOI:** 10.1155/2009/721469

**Published:** 2009-12-21

**Authors:** Nikhil Vasdev, Philippa Holmes, Katie Senior, Philip Haslam, Tahseen Hasan, Trevor Dorkin

**Affiliations:** ^1^Department of Urology, Freeman Hospital, Newcastle upon Tyne NE7 7DN, UK; ^2^Department of Radiology, Freeman Hospital, Newcastle upon Tyne NE7 7DN, UK

## Abstract

We present a rare case of a benign fibroepithelial polyp of the renal pelvis in a patient with familial adenomatous polyposis. In our paper we describe a new minimally invasive technique developed in our unit using an amplatz goose neck snare via a percutaneous nephroscope sheath in the management of the benign fibroepithelial polyp of the renal pelvis and present a current review of management strategies in literature.

## 1. Introduction

Rare benign tumours of the renal pelvis can mimic transitional cell carcinoma and renal cell carcinoma on radiological imaging. We present a rare case of benign fibroepithelial polyp of the renal pelvis in a patient with familial adenomatous polyposis treated successfully in our unit via a minimally invasive percutaneous technique. We also present an up-to-date review of the current literature. 

## 2. Case report

**Figure 1:**
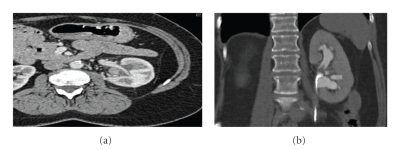
CT Scan findings of fibroepithelial polyp of left kidney.

A 55-year-old woman was referred with acute left loin pain associated with dipstick-positive microscopic haematuria. Intravenous urography showed a filling defect within the left renal pelvis. The filling defect was thought to be a clot. A subsequent CT-Scan (pre- and postcontrast) confirmed the presence of a 13 mm mass arising within the left renal pelvis and extending into the upper pole of the kidney (Figures 1(a) and 1(b)). An urgent left retrograde study with a flexible ureteroscopy confirmed a 13 mm polypoidal lesion within the renal pelvis. Two pinch biopsies from the lesion were taken and histological analysis was representative of either a benign polyp or reactive mucosal hyperplasia. Staining for CK20 showed a normal distribution of umbrella cells. Further levels examined showed focal papillary hyperplasia with no evidence of malignancy. The patients past history included a restorative panproctocolectomy for familial adenomatous polyposis (FAP), total abdominal hysterectomy for uterine fibroids, and excision of a benign fundal gastric polyp. She was a heavy smoker with a 40 pack-year history.

On reviewing the patient's case at our unit's multidisciplinary team meeting a diagnosis of left fibroepithelial polyp of the renal pelvis was established. After discussing surgical strategies a percutaneous nephroscopic excision of the left fibroepithelial polyp of renal pelvis was suggested.

**Figure 2:**
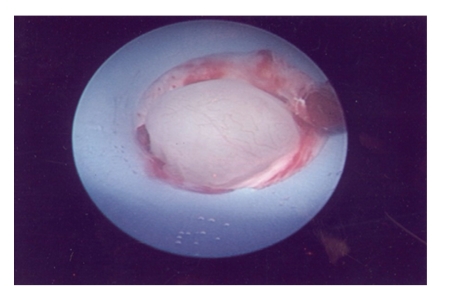
Macroscopic view of fibroepithelial polyp of the kidney within the nephroscopic sheath following application of the 25 mm Amplatz GOOSE NECK Snare to the polyps base.

**Figure 3:**
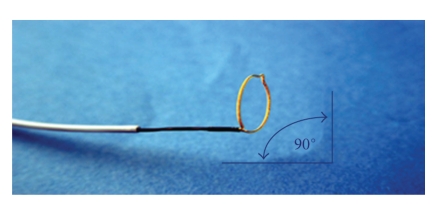
A 25 mm Amplatz GOOSE NECK Snare.

**Figure 4:**
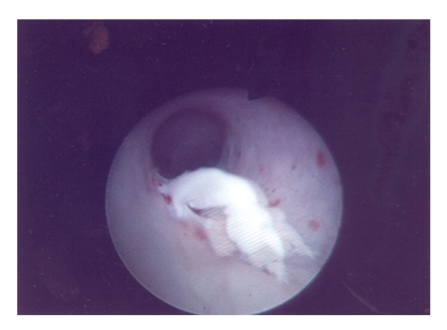
Macroscopic view of base of the fibroepithelial polyp of the kidney following excision with 25 mm Amplatz GOOSE NECK Snare.

The patient underwent a percutaneous nephroscopic removal of the fibroepithelial polyp at 7 months following initial diagnosis. Intraoperatively a retrograde pyelogram was performed which confirmed the presence of the fibroepithelial polyp arising from the infundibulum of the upper pole calyx. The patient was turned prone and the left upper pole calyx was punctured and dilated upto 32 Fr with Amplatz coaxial dilators. A 30 Fr nephroscope was introduced and the fibroepithelial polyp was visualized (Figure 2). A 25 mm Amplatz GOOSE NECK Snare (Figure 3) was deployed across the base of the fibroepithelial polyp and this was subsequently excised in situ (Figure 4). The base of the polyp was fulgurated with Holmium laser (1 Joule × 10 units) via a flexible cystoscope through the nephroscope sheath. A percutaneous drain was removed 24 hours following surgery, and the patient was discharged within 36 hours of surgery. There were no intra/postoperative complications.

Histological analysis of the excised fibroepithelial polyp showed thickening of the lining epithelium. The underlying connective tissue was oedematous and the lesion measured 15 mm macroscopically. There were polypoidal fragments of oedematous stroma lined by reactive urothelium containing a few foci of von Brunn's glands. There was no evidence of malignancy.

**Figure 5:**
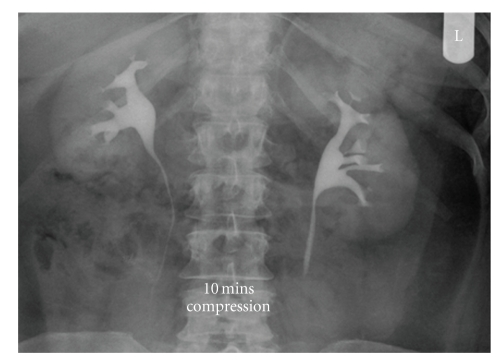
Postoperative intravenous urogram findings confirming the absence of any recurrent fibroepithelial polyp of left kidney.

Postoperative imaging performed 6 months later showed no filling defect within the left renal pelvis or evidence of any new filling defect (Figure 5). The patient has since been discharged and continues to be asymptomatic.

## 3. Discussion

A fibroepithelial polyp of the renal pelvis rarely causes obstruction of the upper urinary tract [1]. The average age of presentation with fibroepithelial polyps in adults is 40 with males predominantly affected [2]. The exact aetiology remains unclear with congenital, obstructive, and traumatic causes being evaluated [3, 4]. Ours is the first case describing a fibroepithelial polyp of the renal pelvis in a patient with familial adenomatous polyposis.

The commonest clinical presentation of fibroepithelial polyps of the renal pelvis is intermittent flank pain (80%) and heamaturia (50%) [5]. Benign fibroepithelial polyps of the renal pelvis arise from benign mucosal projections composed of fibrous stroma lined with surface epithelium [6]. The fibroepithelial polyps of the renal pelvis commonly arise in the pelviureteric junction followed by the posterior urethra and distal/mid ureter [7].

The radiological differential diagnosis for fibroepithelial polyps of the renal pelvis includes blood clot, radiolucent calculi, and transitional cell carcinoma [1, 8]. The usual radiological appearance of a fibroepithelial polyp of the renal pelvis is a slender, filiform, and lucent defect within the pelvicalyceal system. In a large series of fibroepithelial polyp of the renal pelvis none of the lesions were diagnosed on ultrasound [9]. Hence, a CT scan or excretory urogram is required to diagnose the lesion.

The diagnosis of fibroepithelial polyp of the renal pelvis is usually made following nephrectomy or nephroureterectomy for an assumed malignancy [9]. The main features that help distinguish fibroepithelial polyp of the renal pelvis and transitional cell carcinoma include age, radiological characteristics, and anatomical location. Benign fibroepithelial polyps are present in younger patients (30–40 years) and appear as smooth, mobile polypoidal masses in the pelviureteric junction. On the other hand transitional cell carcinoma are seen in older patients (60–70 years) and are fixed, irregular filling defects.

With the advent of modern minimally invasive endourology techniques we recommend percutaneous nephroscopic excision of the histologically proven benign fibroepithelial polyp of the renal pelvis. We recommend a two stage procedure in the management of a benign fibroepithelial polyp of the renal pelvis. The first procedure involves a careful retropyelogram followed by a biopsy of the benign fibroepithelial polyp of the renal pelvis using a flexible ureteroscope. Upon confirmation of the diagnosis the patient is then listed for a second stage percutaneous nephroscopic excision of the benign fibroepithelial polyp. It is essential to delineate the anatomy of the renal pelvis with the benign fibroepithelial polyp using the preoperative images available. With the widespread availability of multiphase CT scanners, allowing imaging during phases of contrast excretion and capacity of coronal reconstruction, CT images may be satisfactory.

Under general anaesthesia a retrograde pyelogram is performed to delineate the anatomy of the pelvicalyceal system. Once the percutaneous puncture is performed and the fibroepithelial polyp is visualized, the base of the poly is identified and a 25 mm Amplatz GOOSE NECK Snare was placed around the base of the fibroepithelial polyp. Using gentle traction the fibroepithelial polyp is then excised in situ. To prevent any recurrence of the fibroepithelial polyp excised we cauterized the base of the polyp using holmium yag laser at a setting of 1 Joule × 10.

Traditionally fibroepithelial polyps of the renal pelvis were treated in the past with a nephroureterectomy and until recently were managed with local resection with primary reanastomosis [9–11]. In current practice minimally invasive percutaneous nephroscopic excision is advocated in order to preserve the affected kidney. We recommend using our percutaneous technique with the use of Amplatz GOOSE NECK Snare to excise the fibroepithelial polyp.

## 4. Conclusion

Renal fibroepithelial polyps are rare lesions of the renal collecting system and should be considered in patients with familial adenomatous polyposis. A cold cup biopsy of the lesion followed by percutaneous nephroscopic excision using an Amplatz GOOSE NECK Snare and laser ablation of the base is an effective management strategy which obviates kidney excision. The possibility of fibroepithelial polyp needs to be considered before subjecting the patient to an unnecessary nephrectomy.
